# Mechanisms of SARS-CoV-2 neutralization by shark variable new antigen receptors elucidated through X-ray crystallography

**DOI:** 10.1038/s41467-021-27611-y

**Published:** 2021-12-16

**Authors:** Obinna C. Ubah, Eric W. Lake, Gihan S. Gunaratne, Joseph P. Gallant, Marie Fernie, Austin J. Robertson, Jonathan S. Marchant, Tyler D. Bold, Ryan A. Langlois, William E. Matchett, Joshua M. Thiede, Ke Shi, Lulu Yin, Nicholas H. Moeller, Surajit Banerjee, Laura Ferguson, Marina Kovaleva, Andrew J. Porter, Hideki Aihara, Aaron M. LeBeau, Caroline J. Barelle

**Affiliations:** 1 Elasmogen Ltd, Liberty Building Foresterhill Road, Aberdeen, AB25 2ZP UK; 2 Department of Pathology and Laboratory Medicine, University of Wisconsin School of Medicine and Public Health, Madison, WI 53705 USA; 3 Department of Cell Biology, Neurobiology and Anatomy, Medical College of Wisconsin, Milwaukee, WI 53226 USA; 4 Molecular and Cellular Pharmacology Training Program, University of Wisconsin School of Medicine and Public Health, Madison, WI 53705 USA; 5 Division of Infectious Diseases and International Medicine, University of Minnesota Medical School, Minneapolis, MN 55455 USA; 6 Center for Immunology, University of Minnesota, Minneapolis, MN 55455 USA; 7 Department of Microbiology and Immunology, University of Minnesota, Minneapolis, MN 55455 USA; 8 Department of Biochemistry, Molecular Biology, and Biophysics, University of Minnesota, Minneapolis, MN 55455 USA; 9 Northeastern Collaborative Access Team, Cornell University, Advanced Photon Source, Lemont, IL 60439 USA; 10 School of Medical Sciences, Scottish Biologics Facility, University of Aberdeen, Foresterhill, Aberdeen, AB25 2ZP UK; 11 Department of Radiology, University of Wisconsin School of Medicine and Public Health, Madison, WI 53705 USA; 12 Carbone Cancer Center, University of Wisconsin-Madison, Madison, WI 53705 USA

**Keywords:** Antibody fragment therapy, Protein design

## Abstract

Single-domain Variable New Antigen Receptors (VNARs) from the immune system of sharks are the smallest naturally occurring binding domains found in nature. Possessing flexible paratopes that can recognize protein motifs inaccessible to classical antibodies, VNARs have yet to be exploited for the development of SARS-CoV-2 therapeutics. Here, we detail the identification of a series of VNARs from a VNAR phage display library screened against the SARS-CoV-2 receptor binding domain (RBD). The ability of the VNARs to neutralize pseudotype and authentic live SARS-CoV-2 virus rivalled or exceeded that of full-length immunoglobulins and other single-domain antibodies. Crystallographic analysis of two VNARs found that they recognized separate epitopes on the RBD and had distinctly different mechanisms of virus neutralization unique to VNARs. Structural and biochemical data suggest that VNARs would be effective therapeutic agents against emerging SARS-CoV-2 mutants, including the Delta variant, and coronaviruses across multiple phylogenetic lineages. This study highlights the utility of VNARs as effective therapeutics against coronaviruses and may serve as a critical milestone for nearing a paradigm shift of the greater biologic landscape.

## Abstract (web summary)

Shark antibodies (Variable New Antigen Receptors, VNARs) are the smallest naturally occurring antibody fragments. Here, the authors screen a VNAR phage display library against the SARS-CoV2 receptor binding domain (RBD) and identify VNARs that neutralize the SARSCoV-2 virus and discuss their mechanisms of viral neutralization.

## Introduction

The COVID-19 pandemic caused by the severe acute respiratory syndrome coronavirus 2 (SARS-CoV-2) has resulted in a devastating global health crisis. Though vaccines are the centrepiece for controlling the pandemic, the benefits of vaccines depend upon complex population vaccination strategies that remain vulnerable to manufacturing or deployment delays. The widely implemented two-dose requirement to achieve efficacy, leaves the possibility of non-compliance for the second dose, a situation that may be exacerbated further by the decision in certain areas to extend the time interval between dosing. The rapid evolution of SARS-CoV-2 into highly infectious variants across the globe also has the potential to impact vaccine efficacy. Researchers have reported that the new SARS-CoV-2 variants can result in reduced sensitivity to antibody therapies, convalescent plasma, and vaccine sera^1–4^. It has been documented that people with compromised immune systems respond poorly to COVID-19 vaccines, thus necessitating the development of additional antiviral therapeutics^5,6^. As we enter the next key stage in our global escape plan from this pandemic, it is vital to develop alternative therapeutic approaches and, concurrently, expand our knowledge of this virus.

Neutralizing antibody (NAb) therapeutics that block virus entry into the host cell have demonstrated efficacy at treating COVID-19 infection. Two NAb therapeutics (LY3819253 and REGN-COV2) received emergency use authorization status from the Food and Drug Administration for use in the clinic^7^. SARS-CoV-2 NAbs target the trimeric spike (S) protein on the viral surface that mediates cell entry. The S protein has two distinct functional subunits that facilitate cell attachment (S1) and fusion of the viral and host cell membranes (S2). The receptor-binding domain (RBD) on the S1 subunit is responsible for engaging angiotensin-converting enzyme 2 (ACE2)—the cognate receptor required for membrane fusion. The RBD exists in two different conformations; the closed “down” conformation and the open “up” conformation which is highly accessible to ACE2. Studies with NAbs that target the RBD have revealed mechanisms of viral neutralization based on changes in the “up” and “down” conformations. In general, NAbs act by blocking the ACE2 binding interface or by trapping the RBD in the unstable “up” conformation. Complicating the development of effective NAbs is the emergence of new SARS-CoV-2 variants with highly mutated S proteins. Studies have shown that mutational changes in the RBD observed in the variants correspond to surface-exposed residues within or proximal to the ACE2 binding interface. These mutations can result in the modification of NAb epitopes leading to attenuated or abrogated neutralization of the virus by antibodies. Thus, there is a need for NAbs that can recognize cryptic epitopes inaccessible to human antibodies that are impervious to mutational drift.

Variable New Antigen Receptors (VNARs) represent an unexplored technology for the development of next-generation NAbs for SARS-CoV-2. VNARs are the smallest (~11 kDa) naturally occurring independent heavy chain-only binding domains in the vertebrate kingdom. Part of the adaptive immune system of sharks, VNARs are evolutionarily distinct from immunoglobulins despite sharing some structural similarity with mammalian heavy and light variable chains. VNARs further differentiate themselves from classical antibodies and single-domain camelid antibodies by lacking a CDR2, but possess the benefits of two additional hypervariable loops (HV2 and HV4), yielding a total of four loops of diversity into their small and simple domain architecture^8^. With characteristic protruding paratopes, the VNARs are pre-disposed to access and bind epitopes not normally available to the planar binding sites of classical human antibodies. This feature allows for the identification of highly potent binders that reach deep into pockets and grooves within the target antigen^9–11^. Furthermore, their amenability to reformatting, cost-effective expression at scale in non-mammalian systems, and exceptional stability in diverse formulation conditions, makes VNARs ideal for clinical translation^12,13^.

Here, we describe the identification and characterization of three monomeric antiviral VNARs (3B4, 2C02, 4C10) that were found to be potent neutralizers of pseudotype and authentic SARS-CoV-2 virus. These VNARs were uniquely potent as monovalent constructs, with efficacies rivalling multimeric variable-heavy-heavy (V_HH_) camelid antibodies and Fc-bound bivalent constructs, including conventional full-length immunoglobulins. The crystal structures of VNARs 3B4 and 2C02 showed markedly different mechanisms of neutralization and underscored the likely resilience of VNARs to SARS-CoV-2 variants. Additionally, the VNARs showed neutralization capabilities against other beta coronaviruses supporting the potential broad therapeutic application of these VNARs and the VNAR platform against both known diseases and future emergent disease. Together the three VNARs described here reinforce the need for the continued expansion of the single-domain heavy-chain only antibody-like drug class.

## RESULTS

### Identification of VNARs against SARS-CoV-2

**Fig. 1:**
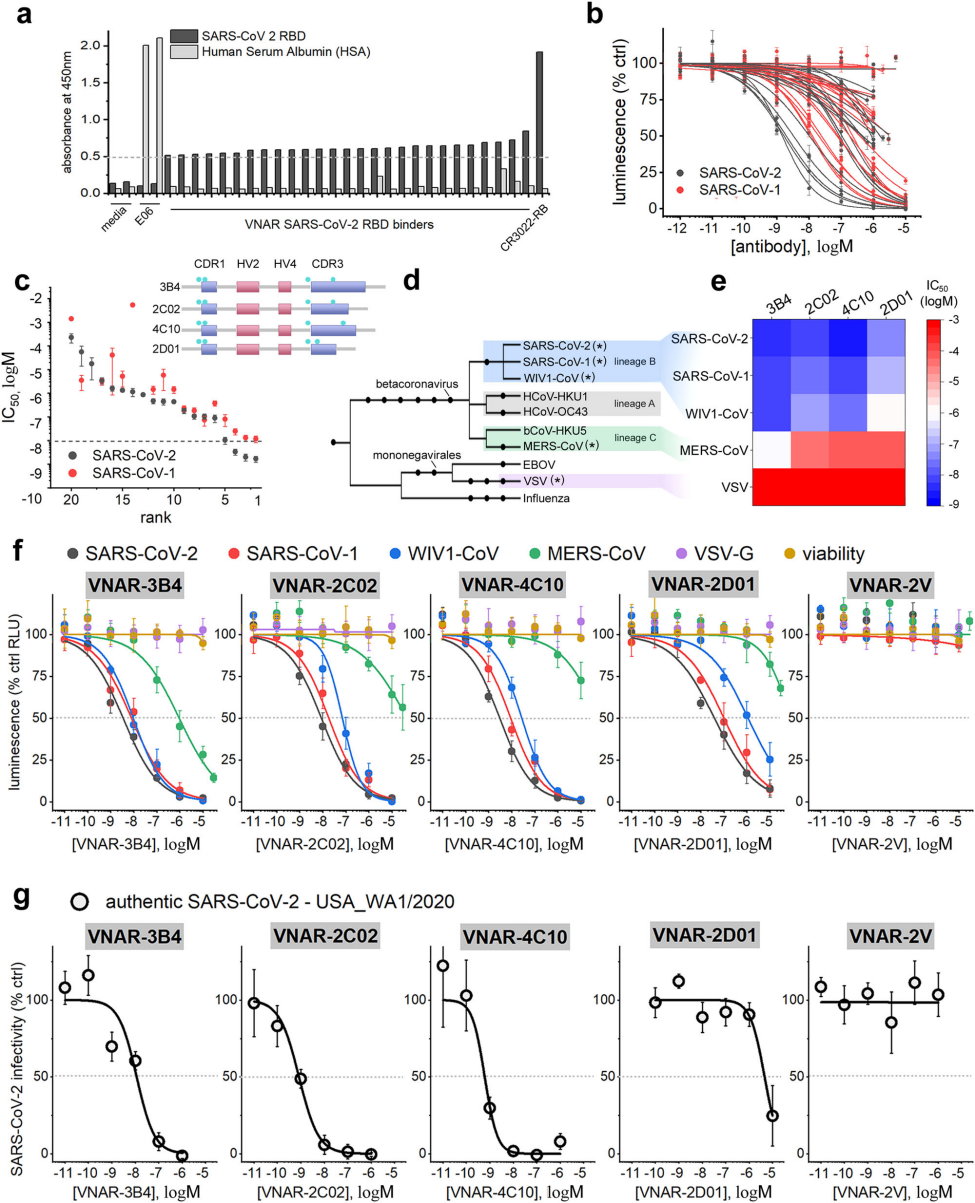
Shark VNARs potently and effectively neutralize the infectivity of multiple coronaviruses. **a** ELISA screen for identification of potential SARS-CoV-2 RBD binders. Negative control wells containing expression media, and positive control wells containing VNAR E06 (anti-serum albumin) and rabbit monoclonal CR3022-RB (anti-SARS-CoV-2 Spike) are indicated. **b** Primary screen for identification of neutralizing VNAR domains. Concentration-dependent neutralization of pseudotyped SARS-CoV-2 (black) or SARS-CoV-1 (red) in HEK293T cells transiently expressing ACE2. Data represents mean ± s.e.m. relative luminescence units (RLUs) from *n* = 3 independent biological experiments. **c** *Left*, rank-ordered IC_50_ values for neutralizing VNARs from panel (**b**). VNARs with IC_50_ < 10 nM (dashed line) were selected for further characterization. Upper right, depiction of primary sequences of selected VNARs, relative length of complementarity determining regions (CDR1, CDR3) and location of cysteine residues (teal) are shown. **d** Phylogenetic tree of selected virus taxa, divergent lineages of betacoronaviruses are shown. Glycoproteins encoded by the indicated viruses (*) were used to generate pseudoviruses. **e** Heatmap summarizing IC_50_ values for neutralization of the indicated pseudovirus with the indicated VNAR antibody. Values are derived from experiments described in (**f**). **f** Secondary validation of selected neutralizing VNAR domains. Concentration-dependent neutralization of viral particles pseudotyped with glycoproteins natively encoded by either SARS-CoV-2 (black), SARS-CoV-1 (red), WIV1-CoV (blue). MERS-CoV (green), or VSV (purple) in Calu-3 cells. Cell viability was also assessed in the presence of increasing concentrations of VNARs (yellow). Data represents mean ± s.e.m. RLU values from *n* = 3 independent biological experiments. **g** Concentration-dependent neutralization of replication-competent authentic SARS-CoV-2, strain USA_WA1/2020 in Vero E6 cells. Data represents mean ± s.e.m. RFU values from *n* = 3 independent biological experiments.

After four rounds of biopanning against the SARS-CoV-2 RBD with the ELSS libraries each containing ~10 billion clones, we isolated nearly two dozen unique VNAR domains that bound to the RBD by ELISA (Fig. 1a). As a primary method for identifying potent inhibitors of viral entry, we screened VNARs in a luciferase-based infectivity assay. Increasing concentrations of VNARs were used to neutralize pseudotyped SARS-CoV-2 and SARS-CoV-1 in ACE2-expressing HEK293T cells using luciferase activity as a readout for viral infectivity (Fig. 1b). As a positive control for our primary screen, we used VHH-72-Fc, a previously identified bivalent single-domain camelid antibody^14^. In our hands, VHH-72-Fc potently neutralized both SARS-CoV-2 and SARS-CoV-1 (IC_50_ = 1.49 nM ± 0.25 nM and IC_50_ = 20.2 nM ± 2.7 nM, respectively), values consistent with the literature. From this screen, VNARs with IC_50_ values below 10 nM were prioritized for further characterization (Fig. 1c). This resulted in the identification of three lead VNARs: 3B4, 2C02, and 4C10. For subsequent controls, we selected a VNAR with relatively low neutralization potency (2D01) and a non-targeting naïve VNAR (2V).

Virus neutralization experiments were next conducted against a series of pseudotyped viruses (Fig. 1D). These secondary validation experiments were performed in Calu-3 cells, an ACE2-expressing human bronchial epithelia cell line that is susceptible to infection by beta-coronaviruses^15^. Our four VNARs were tested for concentration-dependent neutralization of pseudotyped SARS-CoV-2 and SARS-CoV-1 in this cell line. We also screened for cross-reactivity with the pre-emergent ‘SARS-like’ coronavirus, WIV1-CoV, which currently circulates throughout bat populations and displays human tropism via hACE2^16^. Additionally, we assessed neutralization of pseudotyped Middle East respiratory syndrome coronavirus (MERS-CoV), a phylogenetically distant beta-coronavirus which utilizes human DPP4 as a host-cell receptor^17,18^. As a negative control, we screened our panel of VNARs for neutralization of viral particles pseudotyped with vesicular stomatitis virus glycoprotein (VSV-G), an unrelated enveloped virus.

The neutralization potency of the VNARs (3B4, 2C02, 4C10) against pseudotyped SARS-CoV-2 and SARS-CoV-1 was faithfully reproduced in Calu-3 cells and HEK293T-hACE2 cells (Fig. 1d–f, Supplementary Fig. 1 Supplementary Table 1). As expected, VNAR-2D01 displayed ~10-fold lower potency compared to 3B4, 2C02 or 4C10 (Fig. 1d, Supplementary Table 1). High concentrations of non-targeted VNAR-2V had no effect on viral infectivity (Fig. 1f). Each VNAR effectively neutralized viral infectivity of pseudotyped WIV1-CoV suggesting that each VNAR likely binds to epitopes conserved among ‘lineage B’ beta-coronaviruses^17^. In contrast, VNAR-3B4 was uniquely effective at neutralizing cellular entry of viral particles pseudotyped with MERS-CoV spike proteins, a ‘lineage C’ beta-coronavirus, despite sharing only 32% sequence homology with SARS-CoV-2 Spike (Fig. 1d–f)^17^. This observation suggests that VNAR-3B4 likely binds to an interface that is evolutionarily conserved among beta-coronavirus lineages. Importantly, the inhibitory activity observed by these VNAR antibodies was specific to coronaviruses and was not due to deleterious effects on cell health, as demonstrated by the lack of effect on either pseudotyped VSV infectivity or cell viability (Fig. 1e, f).

Finally, we tested the neutralizing efficacy of the VNARs against replication-competent SARS-CoV-2 (strain USA_WA1/2020) in Vero E6 cells (Fig. 1g). In this system, VNAR-3B4 displayed a modest loss of potency (IC_50_ = 11.5 nM ± 5.4 nM), while VNARs-2C02 and 4C10 were both found to be ~10-fold more potent compared to data collected from pseudovirus experiments (IC_50_ = 0.84 nM ± 0.15 nM, and IC_50_ = 0.61 nM ± 0.26 nM, respectively). As expected, 2D01 was the least potent anti-SARS-CoV-2 RBD VNAR antibody (IC_50_ = 4.6 μM ± 1.2 μM), and VNAR-2V failed to have any impact on viral infectivity. IC_50_ values for neutralization of replication-competent SARS-CoV-2 are collated in Table 1. Altogether, these data demonstrate that VNARs 3B4, 2C02, and 4C10 are potent and effective monomeric anti-viral VNARs.

**Table 1:** VNAR IC_50_ values for neutralization of SARS-CoV-2.

VNAR	SARS-CoV-2 USA_WA1/2020
**3B4**	1.15E-8 ± 5.4E–9
**2C02**	8.39E-10 ± 1.5E–10
**4C10**	6.13E-10 ± 2.56E–10
**2D01**	4.60E-6 ± 1.2E–6
**2V**	NA

Half maximal inhibitory concentrations for neutralization of replication-competent SARS-CoV-2 (strain USA_WA1/2020) in Vero E6 cells. Values were calculated from data representing three independent biological experiments. NA, calculated IC_50_ not available.

### Structural basis for SARS-CoV-2 neutralization by VNARs

**Fig. 2:**
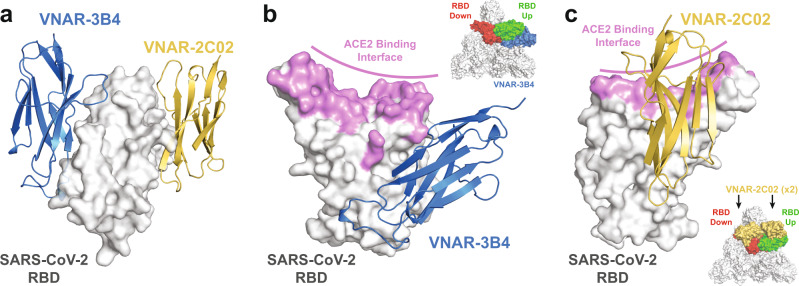
VNARs 3B4 and 2C02 bind to distinct epitopes on the SARS-CoV-2 RBD. **a** Transposed structures of VNAR 3B4 (blue) and 2C02 (yellow) complexes aligned to the RBD colored white as a surface representation. **b** SARS-CoV-2 RBD shown as a surface representation in gray with the ACE2 binding interface colored purple. A cartoon representation of VNAR-3B4 depicted in blue bound to the RBD. Inset shows a surface view of the top of the full-spike trimer with the RBD colored red in the “RBD-down” conformation and colored green in the “RBD-up” conformation with VNAR-3B4 in blue. **c** A 180-degree rotation of the SARS-CoV-2 RBD is shown as in Panel A with a cartoon representation of VNAR-2C02 (yellow) bound to the RBD. Inset shows a surface view of the top of the full-spike trimer with the RBD colored as in Panel A with two VNARs colored in yellow.

**Fig. 3:**
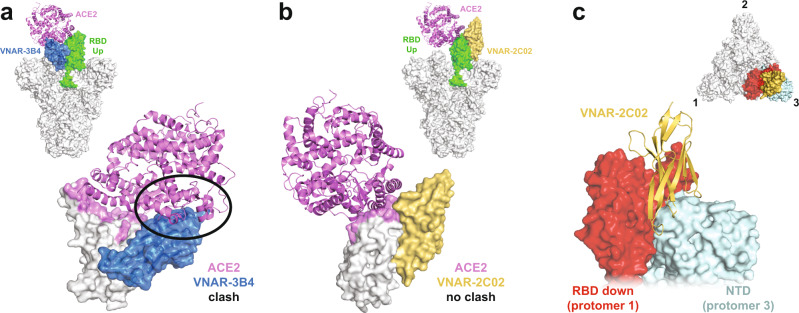
Structural analysis of VNAR 3B4 and 2C02 suggests two unique mechanisms of neutralization. **a** Inset shows the crystal structure of the spike trimer with the RBD in the “up” orientation (green) bound to ACE2 (purple) (PDB ID: 7DF4). An alignment of the RBD (white) from the ACE2 (purple) and the VNAR-3B4 (blue) crystal structures. **b** Inset shows the crystal structure of the spike trimer with the RBD in the “up” orientation bound to ACE2 (purple) (PDB ID: 7BF4). An alignment of the RBDs (white) from the ACE2 (purple) and the VNAR-2C02 (yellow) crystal structures. **c** Figure inset shows the orientation of the main panel with a top-surface view of the spike trimer with numbered protomers and all RBDs in the “down” position (aligned from PDB: 7BF4). The figure shows the VNAR-2C02 structure (yellow) aligned to the “down” RBD from protomer 1 (red) and proximal to the NTD from protomer 3 (cyan).

To understand the mechanism of neutralization, we determined the crystal structure of SARS-CoV-2 spike RBD in complex with VNARs 3B4 and 2C02 at 1.92-Å and 1.96-Å resolution, respectively. The structures show that VNARs 3B4 and 2C02 recognize distinct epitopes on the RBD surface (Fig. 2a, Supplementary Fig. 2), neither of which overlaps with the ACE2 receptor interface. VNAR-3B4 binds distal to the ACE2 binding interface with no direct interaction with the residues involved in ACE2 recognition. This epitope is only accessible to 3B4 when the RBD is in the “up” conformation and is blocked by the N-terminal domains (NTD) of other spike protomers when the RBD is in the “down” position (Fig. 2b, upper inset). Alignment of the 3B4 crystal structure with an available cryo-EM structure of the full spike protein bound to the host receptor ACE2 shows that the framework of 3B4 likely clashes with ACE2 when the RBD is in the “up” conformation. This suggests that the mode of neutralization for 3B4 is through steric occlusion rather than direct competition with the ACE2 binding site (Fig. 3a). This mechanism is similar to Class 1 NAbs as characterized by Barnes, et al, which bind only to “up” RBDs and also block ACE2 binding in some form^19^.

The structure of the RBD in complex with VNAR-2C02 revealed that it binds to the opposite side of the RBD than VNAR-3B4. This epitope is accessible to VNAR-2C02 when the RBD is in the “up’ or “down” conformation (Fig. 2c, lower inset). In contrast to VNAR-3B4, superposition of the VNAR-2C02 crystal structure with the cryo-EM structure of the spike trimer in complex with ACE2 shows that VNAR-2C02 does not come into close contact with ACE2, suggesting that neutralization is not a result of a steric clash with ACE2, but rather through allosteric effects that decrease the population of “up” RBDs that are available to bind ACE2 for viral entry (Fig. 3b). This mechanism is in line with Class 3 NAbs that bind outside of the ACE2 interface^19^. Interestingly, alignment of the VNAR-2C02 structure to an RBD in the “down” conformation in the full-trimer reveals that it binds in a cleft that is formed between the RBD from protomer 1 and the NTD of protomer 3 (Fig. 3c). It is possible that the enhanced efficacy of VNAR-2C02 observed in the viral neutralization assays is a result of additional interactions between VNAR-2C02 and the NTD in this cleft. In this mode, VNAR-2C02 would act to pin the “down” RBD and NTD together and prevent the RBD from sampling the “up” conformation that is necessary for attachment to ACE2, though this remains to be confirmed structurally. This indirect mechanism for blocking ACE2 binding would pair well with other NAbs that directly block ACE2, such as 3B4. The combined neutralization mechanisms of 3B4 and 2C02 would therefore be most therapeutically beneficial when co-administered.

**Fig. 4:**
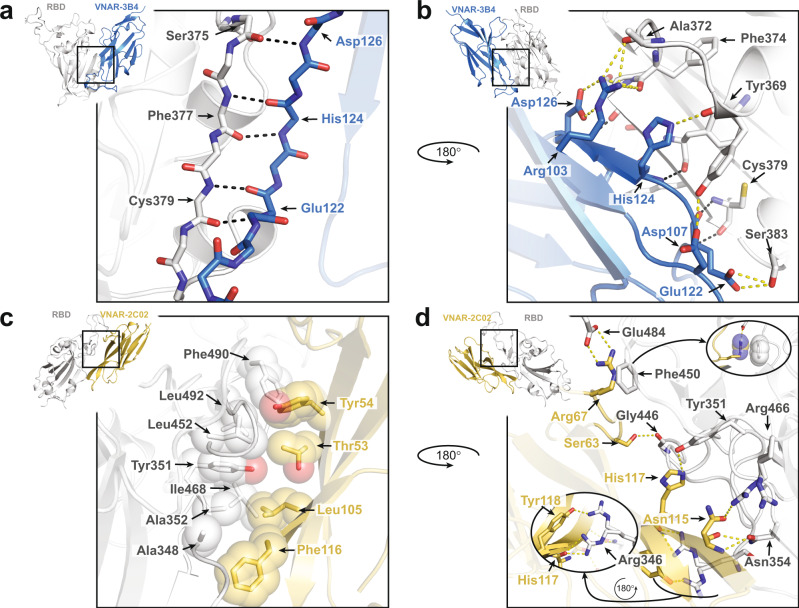
VNARs 3B4 and 2C02 bind the SARS-CoV-2 RBD through uniquely different interactions. **a** Stick representation of the peptide backbone in the primary interaction interface. Interactions between residues Ser-375, Phe-377 and Cys-379 of the RBD (white) and Glu-122, His-124, and Asp-126 of 3B4 (blue) are shown as black dashes. Inset shows the orientation of the zoomed in view. **b** A 180-degree view of the interaction interface. Interactions between residues of the RBD (white) and 3B4 (blue) are shown as yellow dashes with black dashes. Inset shows the orientation of the zoomed in view. **c** Transparent spheres surrounding the stick representations of residues involved in the hydrophobic interactions between the RBD (white) and VNAR-2C02 (yellow). Inset shows the orientation of the zoomed in view. **d** A 180-degree view of the interaction interface between the RBD (white) and 2C02 (yellow). Residues are shown as sticks with yellow dashes representing hydrogen bonds between atoms. Rounded insets show an alternate view of interactions that are partially obscured from view in the main panel. Top inset shows the orientation of the zoomed in view.

The small binding profile of this class of antibody allows VNARs to pack tightly against the RBD and access binding motifs not accessible to conventional antibodies. The primary interaction interface of VNARs 3B4 and 2C02 covers 734 Å^2^ and 792 Å^2^, respectively. Each VNAR covers less area than a standard bivalent antibody, yet still maintains exquisite target specificity. For VNAR-3B4, its interaction was dependent on only 5 residues in the CDR3 region of the VNAR and 7 total residues in the spike RBD. Residues Glu122, His124, Asp126 of CDR3 form an anti-parallel β-sheet with residues Ser375, Phe377, and Cys379 in the β2-strand of the RBD (Fig. 4a). The extensive hydrogen bond (h-bond) pairing of these backbone residues likely accounts for much of the affinity of the interaction, and because it is not dependent on sidechain participation, it makes this part of the interaction primarily residue independent. All three residues of 3B4 that form direct β-sheet interactions, Glu122, His124, and Asp126, also participate in some form of sidechain h-bonding. In addition to the backbone-backbone interactions above, there is also an elaborate h-bond network formed between the terminal amines of Arg103 of 3B4 with the backbone carbonyls of Ala372 and Phe374 that is supported by a side-on intramolecular interaction between Asp126 and Arg103 of 3B4 (Fig. 4b). Another sidechain-backbone interaction occurs between His124 of VNAR-3B4 and the backbone carbonyl of RBD Tyr369. Two sidechain-sidechain h-bonds complete the extent of the interaction – one between Asp107 of 3B4 and Tyr369 of the RBD and a second between Glu122 of VNAR-3B4 and Ser383 of the RBD (Fig. 4b).

The interaction of VNAR-2C02 is dependent on 9 residues separated between the HV2 and CDR3 regions of the VNAR and 12 total residues in the spike RBD. While the 3B4 interaction depended primarily on h-bonding, the interaction of 2C02 largely relies on hydrophobic interactions. Hydrophobic residues Ala348, Ala352, Leu452, Ile468, Phe490, and Leu492, along with the aromatic residue Tyr351, create a nonpolar patch on the RBD which then interfaces with Tyr54, Leu105, and Phe116 of VNAR-2C02 (Fig. 4c). Though Thr53 of VNAR-2C02 is considered polar, the nonpolar methyl group of its side chain appears to be taking part in the hydrophobic core of this interaction. A number of h-bonds help to stabilize the binding of VNAR-2C02 to the RBD (Fig. 4d). RBD residues Asn354 and Arg466 form an h-bond network with Asn115 of 2C02, though the electron density of the crystal structure indicates that multiple rotameric states of Arg466 are possible. Multiple rotamers were also revealed in the density for Arg346 on the RBD indicating that Arg346 transiently h-bonds with Tyr118 or the backbone carbonyl of His117 in VNAR-2C02 (Fig. 4d, oval inset). Arg67 of VNAR-2C02 takes part in two interactions: first, it forms a salt bridge with RBD Glu484, and second, it forms a cation-pi interaction with RBD Phe490. Lastly, Ser63 forms a backbone h-bond with the carbonyl of RBD Gly446.

### VNARs cross-react with closely related coronaviruses

**Fig. 5:**
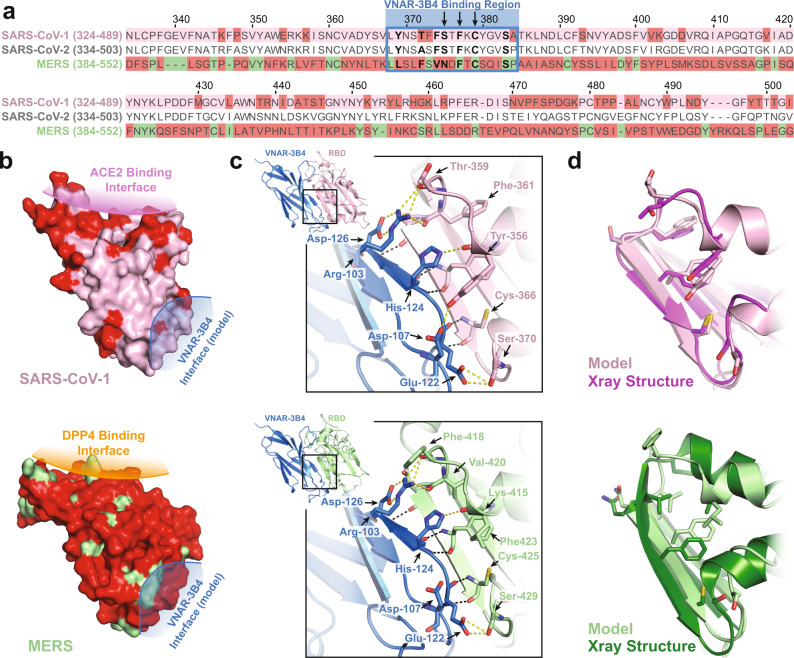
The VNAR-3B4 epitope is highly conserved between closely-related coronaviruses. **a** Sequence alignment between SARS-CoV-1, SARS-CoV-2, and the MERS RBDs. Residues different from SARS-CoV-2 are highlighted in red and the interaction interface for VNAR-3B4 is marked with a blue box. Bolded letters indicate residues critical for the interaction between the RBD and 3B4 with arrows indicating the residues that form a backbone beta-sheet. The sequence alignment is numbered above according to SARS-CoV-2. **b** Surface representations of SARS-CoV-1 (pink, above) and MERS (green, below) with variant residues colored red. The ACE2 binding interface is highlighted in purple for SARS-CoV-1 and the DPP4 binding interface in orange for MERS. The homology-modeled interaction interface for 3B4 is colored blue for both structures. **c** Zoomed in view of the modeled interaction interface between 3B4 (blue) and SARS-CoV-1 (pink, above) and MERS (green, below), with 3B4 colored blue in both pictures. Interacting residues are highlighted as in Fig. 2, showing the backbone interactions in black dashes and sidechain to backbone or sidechain to sidechain interactions shown as yellow dashes. Insets show the orientation of the zoomed in view. **d** Overlays of the 3B4 interface from modeled RBDs and their matching RBDs obtained by x-ray crystallography. The panel above shows the modeled SARS-CoV-1 RBD, colored pink, aligned with the crystal structure of SARS-CoV-1 RBD bound to ACE2 (PDB id: 2AJF), colored magenta. The panel below shows the modeled MERS RBD, colored light green, aligned with the crystal structure of MERS RBD bound to DPP4 (PDB id: 4L72), colored dark green.

In order to understand how our VNARs might bind to the RBD of related coronaviruses, we performed sequence alignments of the three coronaviruses and homology modeling of VNAR-3B4 bound to SARS-CoV-1 and MERS, and VNAR 2C02 bound to SARS-CoV-1. Alignment of the SARS-CoV-1 and SARS-CoV-2 sequences show that there is a high degree of sequence homology between the RBDs, with almost complete conservation in the VNAR-3B4 epitope (Fig. 5a, blue box). A surface view of the RBD shows that most of the sequence variation occurs primarily at the ACE2 binding interface while the VNAR-3B4 interface remains similar (Fig. 5b, top). Among the critical interacting residues, there is a threonine in SARS-CoV-1 in place of the alanine in SARS-CoV-2 of which the backbone carbonyl, and not the sidechain, is the key interactor. Homology modeling of the complex between VNAR-3B4 and the SARS-CoV-1 RBD shows that the critical interactions, including the alanine to threonine change, are likely similar in the SARS-CoV-1:VNAR-3B4 complex (Fig. 5c, top). Crucially, the anti-parallel β-sheet between CDR3 of 3B4 and β2-strand of the RBD as well as the arginine h-bond network are also a key part of the interaction in the model. Alignment of the modeled SARS-CoV-1 RBD with a crystal structure of the SARS-CoV-1 RBD bound to ACE2 (PDB id: 2AJF) shows that the modeling imposed very little structural distortion to align the residues, indicating that only a modest rearrangement of the RBD framework is needed to accommodate binding of 3B4 (Fig. 5d, top).

In contrast to the conservation of the VNAR-3B4 epitope, the VNAR-2C02 epitope is less conserved. Among the 12 interacting residues in SARS-CoV-2, only 5 interactions are conserved in SARS-CoV-1 (Supplementary Fig. 3A, B). Interestingly, homology modeling of the SARS-CoV-1 RBD:VNAR-2C02 complex indicated that similar or replacement interactions take place and that the bulk of the hydrophobic core interactions were intact. Among the 7 residues that are part of this key hydrophobic interaction, four were conserved, two were replaced by residues with similar properties, and only one (L452) is lost (Supplementary Fig. 3A, see arrow annotations). For example, Ala348 has been replaced by Pro335, which is a non-polar like residue when cyclic, and the aromatic residue Phe490 has been replaced by Trp476 (Supplementary Fig. 3C). Conserved residues Tyr338 and Arg453 can still h-bond with His117 and Asn115 of 2C02, respectively. Fortunately, most residues that changed are still able to form similar h-bonds. Among these changes, a variation of Asn354 to Glu341 can still accept an h-bond from the backbone amine but it can no longer donate an h-bond to the carbonyl of the Asn115 of VNAR-2C02. The final major difference between the SARS-CoV-2 structure and the SARS-CoV-1 model is a result of the deletion of a residue equivalent to Glu484. This change results in the loss of an important salt bridge that forms with Arg67 of VNAR-2C02. However, the change of a similar aromatic residue, Phe490 to Trp476, allows Arg67 of VNAR-2C02 to maintain its cation-pi interaction.

Greater variation exists between the SARS-CoV-2 and the MERS RBDs, with only 3 of the 7 interacting residues in the VNAR-3B4 epitope remaining (Fig. 5a, blue box). A surface view of the MERS RBD shows that the sequence variation occurs throughout the RBD (Fig. 5b, bottom). The MERS RBD binds DPP4 instead of ACE2 and a large degree of structural and sequence variation would be expected. Homology modeling of VNAR-3B4:MERS RBD complex found that many of the backbone interactions, including those of the antiparallel β-sheet and arginine h-bond network could still interact (Fig. 5c, bottom). Though the h-bonding between the conserved Ser429 and Glu122 of 3B4 remained, the change from a tyrosine to a leucine certainly eliminates one h-bond formed with Asp107 of VNAR-3B4. It was much less likely that all these interactions remain the same when comparing the modeled MERS RBD to the crystal structure of the MERS RBD bound to its receptor DPP4 (PDB id: 4L72). This alignment has an RMSD of 4.137, indicating that the model and x-ray structure are very dissimilar and that a large structural rearrangement of the MERS RBD framework is needed to fully accommodate the binding of 3B4 (Fig. 5d, bottom). Visual inspection of the alignment shows that the modeling constraints forced both the α1 and α2 helices that surround the β2-strand of the interaction to unwind a single helical turn in order to allow the β2-strand flexibility to interact with 3B4. Such a large structural rearrangement would not be favorable, and it is unlikely that the homology modeling accurately represents the interaction between the MERS RBD and VNAR-3B4. Realistically, the conserved Phe, Cys, and Ser residues of the VNAR-3B4 epitope are the only likely interactions, and the remaining β-sheet and arginine network hydrogen-bonding are lost. Deletion of the h-bonding that occurs between the VNAR and the RBD would explain why VNAR-3B4 weakly binds the MERS RBD and is moderately effective at neutralization of pseudovirus.

### VNARs bind SARS-CoV-2 variants

**Fig. 6:**
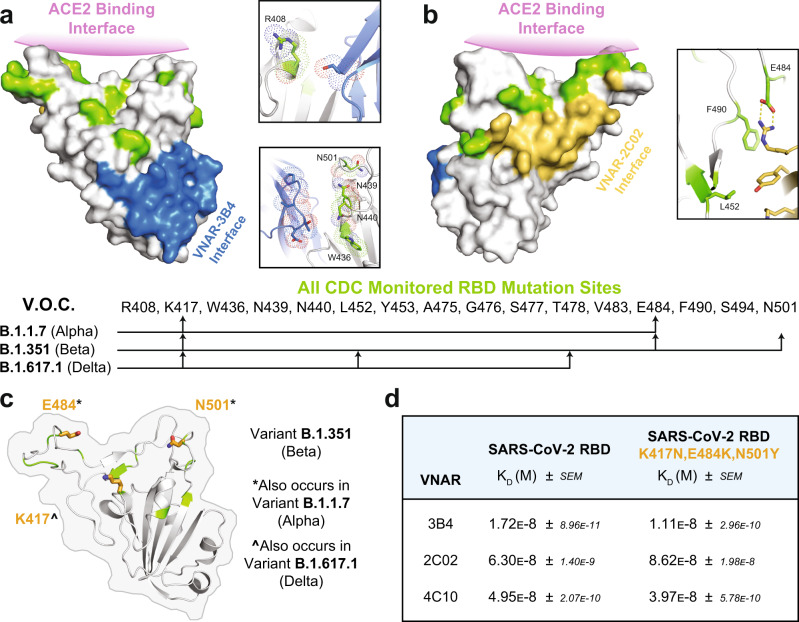
VNAR binding is resistant to emerging RBD mutations. For panels (**a**) and (**b**), all reported mutation sites (as curated by CDC.gov) are colored green in each RBD structure and the ACE2 binding interface is annotated in purple. The list of mutation sites is shown in the center of the figure with arrows drawn to indicate the mutation sites that appear in each of the primary variants of concern (V.O.C). **a** Surface representation of the RBD with the 3B4 binding interface colored in blue. Insets show mutation sites that occur proximal to 3B4, top: (R408) and bottom:(W436, N439, N440, N501). **b** A 180-degree rotation of the RBD with the 2C02 binding interface colored in yellow. Inset shows mutations that are a part of or proximal to the 2C02 interface (L452, E484, F490). **c** A cartoon representation of the RBD with an outline of the surface with mutation sites K417, E484, and N501 displayed as orange sticks. **d** Summary of binding affinities (*K*_D_) for each of the VNARs to the WT SARS-CoV-2 RBD in comparison to the triple mutant RBD indicated in (**c**). Data shown are a result of triplicate experiments using a BLI binding schema with biotin labeled RBDs (see methods).

Emerging mutant strains of SARS-CoV-2 remain a threat to the control of the pandemic. Viral variants are more easily transmissible, and it is uncertain if current vaccine formulations will remain efficacious against them. The CDC is currently monitoring several variants of concern (VOC), including the prominent Alpha and Beta variants that first emerged in the UK and South Africa, respectively, as well as the more recent Delta variant that first appeared in India (Fig. 6). The spike protein is highly susceptible to mutation and many prominent mutations that occur within the RBD alter the interaction with the host ACE2 by enhancing the binding properties (affinity) of the spike protein to ACE2. It is also likely that mutations which affect the ACE2/RBD interface would also diminish or prevent binding of neutralizing antibodies that directly compete at this site.

To visualize the potential influence of RBD mutations on the binding of our VNARs, we mapped mutation sites reported in the growing list curated by the CDC to the RBD of the VNAR-3B4 and 2C02 crystal structures (Fig. 6). Mutation mapping revealed that most of the RBD mutations occur distal to the VNAR-3B4 epitope and are primarily located in the ACE2 binding interface (Fig. 6a). The most proximal mutation sites to VNAR-3B4 (R408, W436, N439, N440, and N501) do not interact with the VNAR, and there are no apparent amino acid changes that would create a clash with VNAR-3B4 at these sites (Fig. 6a, top and bottom insets). This analysis suggests that VNAR-3B4 will not lose its ability to neutralize current SARS-CoV-2 viral variants. Several mutation sites, however, occur directly in the interface of 2C02. Sites L452, E484, and F490 play a direct role in the binding of 2C02, suggesting that 2C02 binding would be altered when binding to viral variants that include these mutation sites (Fig. 6b).

To assess the ability of our VNARs to neutralize variants, we performed biolayer interferometry (BLI) to compare VNAR binding to both WT and mutants RBD (Supplementary Fig. 4). A mutant RBD from the Beta variant, which also contained mutations found in other VOCs, was used (Fig. 6c). Results from the BLI experiments confirmed our initial prediction that these mutations have little to no effect on the affinity of 3B4 (Fig. 6d). Surprisingly, the loss of the salt bridge interaction between VNAR-2C02 and E484 did not have a strong effect on the affinity of VNAR-2C02 (Fig. 6d), further indicating that the VNAR-2C02 interaction is predominantly the result of hydrophobic interactions. While we do not have any structural data for the binding location of VNAR-4C10 on the RBD, the BLI experiments showed that these mutations do not have a substantial effect on VNAR-4C10 affinity as well. Taken together, the mutational mapping analysis and BLI data strongly indicate that the use of VNARs will remain useful neutralizing agents to combat variant strains of the SARS-CoV-2 virus.

## Discussion

Successfully combating the COVID-19 pandemic will require the use of multiple therapeutic modalities rather than reliance on a singular therapeutic tool. Though vaccines will be responsible for controlling disease spread in a majority of cases, vaccines are not a panacea. Research has documented that a subset of individuals is not protected from SARS-CoV-2 infection even after receiving the two dose vaccination regimen and vaccines are not a viable option for immune-compromised individuals^5,20^. In this study, we detail the development of a class of single-domain VNAR binders as neutralizing agents against SARS-CoV-2. Identified by phage display using high-diversity libraries, our lead monomeric VNARs (3B4, 2C02, and 4C10) were able to potently neutralize pseudotype and authentic live SARS-CoV-2 virus at nano and picomolar concentrations. In addition to SARS-CoV-2, the VNARs were able to neutralize closely related coronaviruses, including SARS-CoV-1, and pre-emergent zoonotic viruses (WIV1). One binding domain, VNAR-3B4, was capable of neutralizing coronaviruses across multiple phylogenetic lineages (class 2B and class 2C), owing to partial sequence conservation of the 3B4 epitope. The small size and protruding CDR3 makes 3B4 uniquely effective at accessing this conserved epitope, underscoring the utility of neutralizing VNAR domains. Our data also suggest that the three VNARs we identified would be effective at neutralizing the existing Alpha, Beta, and Delta variants as well as variants yet to emerge. Should vaccination fail due to the emergence of a viral variant, alternative therapies like our VNARS, alone or in combination, are essential to maintaining control over the spread of the virus.

At a molecular weight of approximately 11 kDa, VNARs are smaller than the fragment of antigen-binding domains of human and mouse antibodies (~50 kDa) and even single-domain camelid VHH antibodies (~15KDa). After the initial pseudovirus screen to identify potent neutralizers from our libraries, none of the candidate VNARs underwent affinity maturation and were subsequently characterized as purely monomers. In pseudotype and authentic virus assays, our three lead VNARs performed as well or better than other neutralizing antibodies reported in the literature^21^. Of significance, two VNARs (2C02 and 4C10) had picomolar neutralization IC_50_ values in the authentic virus assay (840 pM and 613 pM respectively). Our VNARs were more effective at neutralization compared to other monovalent single-domain antibodies derived from camelids. In a previous study, investigators found that tethering the affinity matured camelid antibody mNb6 in triplicate to form a trimer (mNb-tri) resulted in a highly potent construct with low picomolar neutralization IC_50_ values against both pseudo and authentic SARS-CoV-2 virus. Remarkably, the IC_50_ values of this trivalent construct were only one order of magnitude lower than our monovalent VNAR-4C10. As a positive control in our initial VNAR screen we used VHH-72-Fc, a bivalent form of the camelid VHH-72. Though not effective at neutralizing pseudovirus as monomer, VHH-72-Fc had a low nanomolar potency (1.5 nM) which was only slightly better than our best VNAR (2C02 - 2.8 nM). These findings highlight the ability and versatility of the diminutive VNAR scaffold for the development of highly specific and effective agents against a given target.

Not only do VNARs 3B4 and 2C02 bind the SARS-CoV-2 RBD at different epitopes, but their primary modes of interaction were also completely different. Analysis of the crystal structures revealed that VNAR-3B4 relied heavily on hydrogen bonding while hydrophobicity was at the core of VNAR-2C02 binding to the RBD. Interestingly, only two of the interactions in VNAR-3B4 were dependent on RBD residue identity, as the majority of hydrogen bonding occurred in direct contact with the peptide backbone of the spike RBD. We also found that VNAR-3B4 bound the RBD solely via its CDR3, the canonical VNAR paratope. In the case of VNAR-2C02, 7 of the 12 interface residues of the RBD have hydrophobic qualities driving the interaction. This feature also makes the 2C02 interaction residue independent to the extent that residue identity of this interface maintains its non-polar features. In addition to binding through the CDRs, the interaction between VNAR-2C02 and RBD was supported by two residues in the HV2 region, Ser63 and Arg67, providing additional evidence for the functional significance of the HV2 domain. The partial residue independence of each interaction indicates that VNARs 3B4 or 2C02 as a treatment could maintain potency for binding the RBD in emerging spike mutants or other coronaviruses. This independence is further illustrated by the BLI experiment with VNAR-2C02 and the Beta variant RBD. Although Arg67 of VNAR-2C02 forms a salt bridge with E484 of the wild type RBD, loss of that salt bridge in the variant RBD did not have a significant effect on VNAR binding.

Comparison of the VNARs to other NAbs in the literature reveals distinct differences about their epitopes and mechanisms of action. VNAR-3B4 binds an epitope on the RBD that is highly conserved between SARS-CoV-1 and SARS-CoV-2 that is distal to the ACE2 binding interface. The VNAR-3B4 epitope partially overlaps with other NAbs reported in the literature including the human IgGs CR3022 and EY6A (Supplementary Fig. 5A)^22–24^. While CR3022 and EY6A destabilize spike protein by trapping the RBD in the “up” conformation, they do not result in a direct steric clash with ACE2 like VNAR-3B4. VHH-72 binds near the VNAR epitope and makes similar backbone interactions to VNAR-3B4 (Supplementary Fig. 5B)^14^. VNAR-3B4 and VHH-72 bind to the RBD on opposite sides of the conserved fold resulting in binding modes that are quite distinct. VNAR-2C02 likely neutralizes by pinning the RBD in the down position, thus blocking access to ACE-2. Antibodies Fab 2–4 and BD23 neutralize through a similar mechanism, however, there is minimal epitope overlap with VNAR-2C02 (Supplementary Fig. 6A)^19,25^. There is epitope overlap between VNAR-2C02 and Nanosota-1, a neutralizing VHH that was recently identified from our nanobody library. VNAR-2C02 and Nanosota-1 can both bind the RBD in down conformation, but Nanosota-1 directly blocks ACE2 binding and also recognizes a number of mutational prone residues in the ACE2 binding interface (Supplementary Fig. 6A)^26^. There is partial epitope overlap of VNAR-2C02 with other NAbs, however, those antibodies act through ACE2 antagonism rather than through a potential bivalent RBD-NTD interaction that pins down the RBD^7^. Importantly, the separate epitopes of 3B4 and 2C02 suggest that they would be therapeutically effective as a multi-NAb cocktail, similar to the REGEN-COV antibody cocktail of casirivimab and imdevimab, which bind to closely located epitopes. Taken together, our VNARs occupy a unique molecular space that have potential to completely alter the landscape of biologics by making antibody-class drugs even smaller, yet as potent and efficacious.

## Methods

### Plasmids

Plasmids used for generating pseudovirus stocks were sourced as follows: plasmid encoding an *Env*-defective, luciferase-expressing HIV-1 genome (pNL4-3.luc.R-E^−^)^27,28^ was obtained through the NIH AIDS Reagent Program; plasmids encoding SARS-CoV-1 and SARS-CoV-2 Spike were from Fang Li (Addgene plasmid #145031 and #145032, respectively)^29^; WIV1-CoV Spike was from Alejandro Balazs (Addgene plasmid #164439)^30^; MERS-CoV Spike was from Sino Biological (VG40069-NF); and VSV-G was from Bob Weinberg (Addgene plasmid #8454).

### Cell culture

HEK293T cells (human embryonic kidney), Vero E6 cells and Calu-3 cells were purchased from ATCC. HEK293T cells stably overexpressing human angiotensin-converting enzyme 2 (HEK293T-hACE2) were sourced from BEI Resources (NR-52511). All cells were maintained in DMEM supplemented with 10% fetal bovine serum, 100 units/ml penicillin and streptomycin, 292 µg/ml l-glutamine and cultured at 5% CO_2_ and 37 °C. All cell culture reagents were purchased from Thermo Fisher.

### Recombinant SARS-CoV-2 RBD

Recombinant RBD expressed in Chinese hamster ovary cells was generous gift from Fang Li, PhD (University of Minnesota, Department of Veterinary and Biomedical Sciences) and was also purchased from R&D Systems (cat #10534-CV). Prior to biotinylation, ~0.5 mg of the RBD in 20 mM Tris-HCl, 200 mM NaCl was dialyzed against phosphate-buffered saline (PBS, pH 7.4) for 4 h using Slide-A-Lyzer™ cassettes, 10 K MWCO (Thermo Scientific) to remove tris-base. Biotinylation (1–3 biotins per RBD molecule) was achieved using EZ-Link Sulfo-NHS-LC-Biotinylation Kit (Thermo Scientific) following manufacturer’s instructions, and a final dialysis against PBS, pH 7.4 to remove excess biotin. All phage display selection rounds were conducted with biotin-RBD, while screening (including binding ELISA) was conducted with non-biotinylated RBD protein. For the crystal structure in complex with VNAR 2C02, RBD expressed in insect cells was used.

### Identification of SARS-CoV-2 VNARs

Elasmogen’s proprietary next-generation synthetic, multi-framework VNAR libraries (ELSS) were constructed by combining naïve VNAR frameworks, varying CDR3 lengths, inclusion of sequence diversity within CDR1 and CDR3, and in some cases, incorporation of non-canonical cysteine residues in CDR1 and CDR3. These closely related libraries represent either type II or IIb (also referred to as type IV) VNARs^9,10^. VNAR binders to the SARS-CoV-2 RBD were isolated from these ELSS libraries. The libraries were maintained in Elasmogen’s proprietary phagemid vector with a diversity of ~2 × 10^10^ unique clones. Each library was inoculated into 2X TY growth media supplemented with 1% glucose (w/v), 100 µg/mL ampicillin (2X TY-Amp/Glu) and grown at 37 °C to mid-log phase (OD_600_ of 0.45–0.6) prior to infection with M13K07 helper phage for 30 min in a static 37 °C incubator to obtain a phage rescued library of VNAR-presenting phage. This infected culture was re-suspended in fresh 2X TY media supplemented with 100 µg/mL ampicillin, 50 µg/mL kanamycin (2X TY-Amp/Kan) and incubated overnight at 30 °C on a shaking platform. Phage was PEG-precipitated from the culture supernatant and used for round one of bio-panning. The phage VNAR library, was panned against biotin-RBD captured on Dynabeads™ M-280 Streptavidin (Thermo Fisher Scientific). Library phage and Dynabeads™ were pre-blocked (3% (w/v) milk in PBS - MPBS) for 1 h, at room temperature on a disc rotator. Biotin-RBD (400 nM) was added to blocked Dynabeads™, incubated for 1 h, rotating at room temperature. Non-specific phage were deselected from the library by incubating with blocked beads for 1 h at room temperature. Biotin-RBD (~400 nM) decorated Dynabeads™ were incubated with deselected phage for 1 h, rotating at room temperature (round 1). Beads were washed 5X with PBST and PBS before eluting with 100 mM triethylamine and neutralized by adding 1 M Tris-HCl pH 7.5. Mid-log phase *E. coli* TG1 cells were infected with eluted phage for 30 min, at 37 °C, and plated on 2X TY agar plates supplemented with 1% glucose and 100 µg/mL ampicillin. Three additional rounds of selection were conducted using the same antigen concentration, and wash stringency. After each round of panning, 90 individual clones were picked and inoculated into 2X TY-Amp/Glu and were grown overnight at 37 °C, with shaking at 220–250 rpm. The next day, these overnight cultures were used to inoculate 2X TY-Amp and infected with M13 helper phage to obtain populations of monoclonal VNAR-presenting phage. Enrichment of antigen binding monoclonal phage was evaluated using a direct antigen-binding ELISA.

### ELISA

The 96 wells of F-bottom microtitre plates (Greiner Bio-One) were coated with 1 µg/mL SARS-CoV-2 RBD protein in PBS, pH 7.4 for 1 h at 37 °C. The plates were blocked with 4% MPBS, incubated for 1 h at 37 °C. Blocked plates were washed 3X with PBST (PBS + 0.1% (v/v) Tween-20) and PBS. Supernatant of individual monoclonal phage was added to designated wells, incubated for 1 h at room temperature before washing 3X with PBST and PBS. Binding was detected using 1:10,000 dilution of anti-M13-horseradish peroxidase (HRP)-conjugated (Stratech Scientific) prepared in 3% (w/v) MPBST. Anti-SARS-CoV-2 RBD antibody CR3022-RB (1 µg/mL). After washing, 100 µL of TMB substrate (Fisher Scientific) was added, neutralized with 50 µL 1 M H_2_SO_4_ and absorbance measured at 450 nm with a microplate absorbance reader.

### Production of SARS-CoV-2 VNARs

Individual and unique, by DNA sequence, positive binders to RBD protein were identified for further characterization. Glycerol stocks of the selected VNAR domains were used to inoculate 2X TY-AG, incubated at 37 °C for 6–8 h, shaking at 250 rpm. The culture was centrifuged, and the cell pellet resuspended in fresh Terrific Broth (TB) media supplemented with phosphate salt, 1% (w/v) glucose and 100 µg/mL ampicillin, incubated overnight at 37 °C at 250 rpm. The overnight culture was centrifuged, re-suspended in fresh TB media supplemented with phosphate salt, and 100 µg/mL ampicillin, incubated at 25–28 °C, 200 rpm for 1 h. Protein expression was induced for 6 h with a final concentration of 1 mM isopropyl β-D-1-thiogalactopyranoside (IPTG) added to the culture, incubated at 25–28 °C, 200 rpm. After 6 h induction, the culture was centrifuged and the resulting cell paste was re-suspended with ice-cold 2-[Tris-(hydroxymethyl)-methylamino]-1-ethane sulfonic acid (TES) by incubation for 15 min at 15–20 °C, on a 100-rpm shaking platform. An equal volume of ice-cold 2.5 mM MgSO_4_ was added to the culture, and the incubation continued for a further 15 min. The culture was centrifuged for 30 min, at 4 °C, and the supernatant carefully collected. The supernatant was filtered through a 0.22 µm filter to further clarify the protein solution prior to purification via a poly-histidine tag using immobilized metal affinity chromatography (IMAC). 2C02 used in crystallographic analysis was expressed in SHuffle T7 express *E. coli* strain (NEB) and purified using IMAC and size-exclusion chromatography.

### Production of pseudotyped viruses

Pseudotyped retroviruses expressing a luciferase reporter gene were prepared by co-transfecting HEK293T cells with a plasmid encoding an Env-defective and luciferace-encoding HIV-1 genome (pNL4-3.luc.R-E-) and a plasmid encoding either SARS-CoV-2 Spike, SARS-CoV-1 Spike, WIV1-CoV Spike, MERS-CoV Spike, or vesicular stomatitis virus glycoprotein (VSV-G). Transfections were performed with 15 µg of each plasmid in a 175 cm^2^ culture flask (Nunc) using Lipofectamine 3000 (Thermo Fisher). Medium-containing transfection complexes was replaced with fresh complete medium after 8 h. Secreted pseudovirus particles were harvested from HEK293T supernatant 72 h after transfection. Pseudovirus stocks were stored at −80 °C in single-use aliquots and were titered using the Reed-Muench method to determine TCID_50_ concentrations^31^.

### Pseudovirus neutralization assays

Cell infection assays were carried out as described previously^32^. HEK293T-hACE2 or Calu-3 cells were used to monitor pseudovirus infectivity. The day before viral transduction, 1 × 10^4^ HEK293T/ACE2 cells or 5 × 10^4^ Calu-3 cells were plated into each well in 96-well plates (Midwest Scientific). The day of transduction, 10-fold serial dilutions of VNARs were prepared in a separate 96-well plate in complete medium and supplemented with a 1000xTCID_50_ dose of pseudovirus per well, the VNAR/pseudovirus mixture was incubated for 1 h at 37 °C. Media was removed from cultured cells and replaced with the pre-incubated VNAR/pseudovirus mixture, plates were incubated overnight at 37 °C and 5% CO_2._ The following day, cells were washed twice with DPBS and media was replaced with a complete medium. Three days post-transduction, cells were assayed for luciferase activity using ONE-Glo Luciferase Assay System (Promega). Briefly, culture media was partially removed, leaving 50 µl of media/well, an equal volume of ONE-Glo reagent was added to each well, plates were incubated for 3 min at room temperature on a microplate shaker, and aliquots from each well were transferred to a solid white 96-well assay plate (CellStar). Luminescence was measured using a Tecan Infinite M1000 microplate reader and Magellan Standard v7.2 software. Luminescence values are reported relative to levels measured in cells treated with virus alone, background corrected by luminescence values in cells unexposed to virus. Samples were tested in technical triplicates across three independent biological experiments. Data were processed in Microsoft Excel v2110. Concentration-response curves and IC_50_ values were generated in OriginLab 2021b.

### Authentic SARS-CoV-2 neutralization assays

Vero E6 cells (2.5 × 10^4^) were seeded in each well of a 96-well black/clear flat bottom TC-treated plate (Falcon) and incubated in DMEM overnight at 37 °C with 5% CO2 before infection. Antibody samples were serially diluted in DMEM and incubated with mNeonGreen SARS-CoV-2 at 37 °C for 1 h^33^. Medium was removed from cells and the virus-antibody mixture was added to achieve a final multiplicity of infection of 0.1 plaque-forming units per cell. The cells were incubated at 37 °C with the virus-antibody mixture for 24 to 26 h. After incubation, cells were fixed in 4% paraformaldehyde at 4 °C for 30 min. The paraformaldehyde-virus-plasma mixture was removed, cells were washed once with PBS, and 50 μL of PBS was added to each well. The fluorescence signal was determined by reading the plates on a Synergy H1 hybrid multimode reader (BioTek) with BioTek Gen5 v2.09.1 software, using excitation/emission wavelengths of 488/517 nm. Percent maximal infection was determined for each dilution as the ratio of the fluorescent signal to the maximal signal for non-treated controls in the same plate. Each sample was tested with two technical replicates across three independent biological experiments. Data were processed in Microsoft Excel v2110. Concentration-response curves and IC_50_ values were generated in OriginLab 2021b.

### Biolayer interferometry (BLI)

BLI measurements were obtained using a forteBIO OctetRED96e instrument. Biotinylated S-RBD protein (AcroBiosystems) was captured on a SAX (high precision streptavidin) biosensor. The SAX biosensors were hydrated in an assay buffer of PBS containing 0.1% BSA. Hydrated SAX sensors were equilibrated for 30 s in assay buffer before the S-RBD protein was loaded for 45 s. Following the bait protein capture a second equilibration step was performed for 60 s. Serial dilutions of the anti-SARS-CoV-2 VNARs (1:2 dilutions from 1 µM to 31.25 nM) were injected over the biosensor for 240–300 s followed by 240–300 s dissociation. Two controls were included in each measurement, a no bait (S-RBD) control was used that was exposed to the 1 µM concentration to ensure the VNAR did not bind to an empty SAX sensor and a no VNAR control was used to ensure additional signal on the S-RBD loaded sensor was not non-specific. Binding affinities were determined by analysis of generated binding curves in the Octet DataAnalysis v12.0.2.3 software program. The controls were averaged and subtracted from the data before modeling and the dissociation constant (*K*_D_) was calculated. Figures were generated by plotting data in GraphPad Prism v9.0.

### VNAR-RBD crystallization

Purified SARS-CoV-2 RBD and VNARs were mixed at ~1:1 molar ratio and the complex formed was isolated using a Superdex200 size-exclusion chromatography (SEC) column operating with 10 mM Tris-HCl (pH 7.4) and 150 mM NaCl. The isolated complex was concentrated by ultrafiltration to ~11 mg ml^−1^ and subjected to crystallization. The RBD-VNAR 3B4 complex was crystallized by the sitting drop vapor diffusion method, using a reservoir solution containing 0.2 M sodium acetate and 20% (w/v) PEG 3350. The crystals grew to full size in three days. The RBD-VNAR 3B4 crystals were cryo-protected by brief soaking in the reservoir solution supplemented with 25% ethylene glycol and flash-cooled by plunging in liquid nitrogen. X-ray diffraction data were collected at the Advanced Photon Source NE-CAT beamline 24-ID-C. The RBD-VNAR 2C02 complex was prepared similarly to the above, except that the NaCl concentration of 200 mM was used in SEC and the isolated complex was concentrated to ~15 mg ml^−1^. The RBD-2C02 complex was crystallized by the sitting drop vapor diffusion method, using a reservoir solution containing 0.2 M sodium sulfate and 20 % (w/v) PEG 3350. The RBD-2C02 crystals were cryo-protected as above and data collected at the NE-CAT beamline 24-ID-E. All X-ray diffraction data were processed using XDS version 20210205^34^. The RBD of the spike protein of SARS-CoV-2 (PDB ID: 6YM0) and shark IgNAR variable domain (PDB ID: 4HGK) were used as molecular replacement search models. Molecular replacement calculation using PHASER version 2.8 identified the solution with two copies each of RBD and VNAR 3B4 in the asymmetric unit^35^. Iterative manual model building and refinement were done using COOT version 7766 and PHENIX version v.1.19.2-4158, respectively^36,37^. The mFo-DFc map clearly showed the presence of glycans in later stages of the refinement. The glycan on Asn343 was manually modeled into the mFo-DFc density using COOT. Molecular replacement for the RBD-2C02 complex was done using the RBD and VNAR 3B4 portions of the RBD-3B4 structure separately as search models. Refinement and model building procedures were similar to those for the RBD-VNAR 3B4 complex. In the RBD-2C02 crystal there is one complex in the asymmetric unit. The summary of data collection and model refinement statistics is shown in Supplementary Table 2. The coordinates and structure factors have been deposited in the Protein Data Bank with the accession code 7SPO and 7SPP.

### Sequence alignment and homology modeling

SARS-CoV-1, SARS-CoV-2, and MERS sequence alignments were generated using published sequence data in the alignment program Clustal X2. The resulting alignments were used in the “Target-Template Alignment” tool in the online modeling program, SwissModel. The x-ray structures of the RBD-VNAR 3B4 and 2C02 complexes were used as templates for modeling the SARS-CoV-1 and MERS RBD-VNAR complexes. By default, SwissModel will only account for one of the protein chains in a coordinate file and will not effectively model interactions between residues of multiple chains. To circumvent this, coordinate files for the RBD-VNAR complexes were altered in PyMol version 2.5 to include both protein chains in a single segment and single chain. The sequence was renumbered in numerical order and thirty amino acids of the pattern Gly-Ala-Ser were inserted as a spacer between the proteins in the modified sequence. The modified coordinate file was exported to be used as the template for the “User Template” tool in SwissModel. Text sequences for SARS-CoV-1 and MERS RBD-VNAR complexes, organized in a similar fashion as the template coordinate file and containing the G-A-S insertion, were uploaded and the model was run. In PyMol, resulting models were then separated into individual chains and segments and renumbered to match the original coordinate file. Homology of the modeled RBD-VNAR interactions were compared to the original coordinate files.

### Data statistics

All infectivity data represents the mean ± s.e.m. of at least three independent biological replicates. No data exclusions were made. No randomization or blinding measures were made.

### Reporting summary

Further information on research design is available in the Nature Research Reporting Summary linked to this article.

## Supplementary information

Supplementary Information

Reporting Summary

## Data Availability

The data that support this study are available from the corresponding authors upon reasonable request. The structural data generated in this study have been deposited in the RCSB Protein Data Bank. Structures of SARS-CoV-2 RBD in complex with VNAR 3B4 or VNAR 2C02 are original to this work and can be found with accession codes 7SPO and 7SPP, respectively. Previously reported structures retrieved from PDB: 2AJF, 4L72, 6YM0, 4HGK, 7DF4, and 7BF4. Sequences of lead VNAR antibodies are collated in Supplementary Table 3. Source data are provided with this paper.

## Source data

Source Data
