# Supplementary material for: Mechanisms of SARS-CoV-2 neutralization by shark variable new antigen receptors elucidated through X-ray crystallography
Source: Nat Commun. 2021 Dec 16;12:7325. doi: 10.1038/s41467-021-27611-y (PMC8677774; doi:10.1038/s41467-021-27611-y)
Supplement: Supplementary file 1 — Supplementary Information [file 41467_2021_27611_MOESM1_ESM.pdf]

Supplementary Information

Supplementary Table 1 - VNAR IC_{50} values for pseudovirus neutralization. Half maximal inhibitory concentrations for neutralization of pseudotyped SARS-CoV-2, SARS-CoV-1, WIV1-CoV, MERS-CoV, and VSV in Calu-3 cells and HEK293T-hACE2 cells. Values were calculated from data representing 3 independent experiments. NT, not tested. NA, calculated IC_{50} not available.

Cell line	VNAR	SARS-CoV-2	SARS-CoV-1	WIV1-CoV	MERS-CoV	VSV
Calu-3	**3B4**	3.44E-9 ± *8.5E-10*	7.93E-9 ± *1.8E-9*	9.49E-9 ± *1.8E-9*	1.05E-6 ± *2.3E-7*	NA
	**2C02**	8.67E-9 ± *2.2E-9*	1.82E-8 ± *5.2E-9*	7.11E-8 ± *2.5E-8*	5.02E-5 ± *3.7E-5*	NA
	**4C10**	3.02E-9 ± *7.4E-10*	8.95E-9 ± *2.3E-9*	2.72E-8 ± *3.9E-9*	1.88E-5 ± *9.6E-5*	NA
	**2D01**	3.92E-8 ± *9.3E-9*	1.01E-7 ± *2.25E-8*	1.13E-6 ± *2.4E-7*	6.36E-5 ± *4.14E-5*	NA
	**2V**	NA	NA	NA	NA	NA
HEK293T-hACE2	**3B4**	2.92E-9 ± *7.5E-10*	6.62E-9 ± *1.8E-9*	5.62E-9 ± *9.8E-10*	NT	NA
	**2C02**	7.71E-9 ± *1.8E-9*	1.48E-8 ± *2.7E-9*	7.13E-8 ± *2.5E-8*	NT	NA
	**4C10**	5.34E-9 ± *2.9E-9*	8.95E-9 ± *2.3E-9*	2.73E-8 ± *5.8E-9*	NT	NA
	**2D01**	5.37E-8 ± *1.9E-8*	8.52E-8 ± *1.8E-8*	1.14E-6 ± *2.4E-7*	NT	NA
	**2V**	NA	NA	NA	NT	NA

Supplementary Table 2 - Data collection and refinement statistics.

**Data Collection**	**RBD_VNAR 3B4**	**RBD_VNAR 2C02**
Wavelength	0.979	0.979
Resolution range	72.96 - 1.92 (1.99 - 1.92)	40.1 - 1.96 (2.03 - 1.96)
Space group	$P2_12_12_1$	$P2_122_1$
Unit cell		
a, b, c (Å)	81.14 90.74 122.69	61.45 74.23 75.46
Total reflections	474654 (47304)	99477 (9773)
Unique reflections	69764 (6868)	25060 (2468)
Multiplicity	6.8 (6.9)	4.0 (3.9)
Completeness (%)	99.79 (99.75)	97.93 (98.44)
$<I/\sigma(I)>$	20.06 (1.10)	11.52 (1.40)
R_{merge}	0.06929 (1.538)	0.05812 (0.8962)
R_{meas}	0.075 (1.661)	0.06726 (1.037)
R_{pim}	0.02841 (0.6241)	0.03286 (0.5072)
$CC_{1/2}$	0.998 (0.543)	0.998 (0.856)
**Refinement**		
Reflections for R_{work}	69703 (6863)	24959 (2456)
Reflections for R_{free}	3510 (390)	1263 (108)
R_{work}	0.1901 (0.3179)	0.1807 (0.3702)
R_{free}	0.2270 (0.3487)	0.2206 (0.4016)
No. of non-H atoms	5273	2587
Macromolecules	4917	2434
Ligands	230	49
Solvent	744	390
Protein residues	625	305
R.m.s. deviations		
Bond length (Å)	0.014	0.012
Bond angles (°)	1.11	0.99
Ramachandran plot		
Favored (%)	97.07	96.35
Allowed (%)	2.93	3.65
Outliers (%)	0.00	0.00
Average B-factor	61.79	55.79
Macromolecules	61.25	55.81
Ligands	93.75	87.86
Solvent	58.67	49.74
PDB ID	7SPO	7SPP

Statistics for the highest-resolution shell are shown in parentheses.

Supplementary Table 3 – Amino acid sequences of neutralizing VNAR antibodies.

VNAR ID	VNAR sequence
**3B4**	ASVNQTPRTATKETGESLTINCVVTGARCGLSRTSWFRKNPGTTDWERMSIGGRYVESVNKGAKSFSLRIKDLTVADSATYICRAWSDTSQKPCHAWEQKMWEGHVDGAGTVLTVNQASGAHHHHHHGAEFEQKLISEEDL
**2C02**	ASVNQTPRTATKETGESLTINCVVTGASCSWSRTYWYRKNPGSSNQERISISGRYVESVNKGAKSFSLRIKDLTVADSATYYCKALINTGKDCTMNFHYDGAGTVLTVNQASGAHHHHHHGAEFEQKLISEEDL
**4C10**	ASVNQTPRTATKETGESLTINCVVTGAKCGWSGTSWFRKNPGTTDWERMSIGGRYVESVNKGAKSFSLRIKDLTVADSATYICRAIPHRRWWNNIDRCEFDFDGAGTVLTVNQASGAHHHHHHGAEFEQKLISEEDL
**2D01**	ASVNQTPRTATKETGESLTINCVVTGASCAWSRTYWYRKNPGSSNQERISISGRYVESVNKGAKSFSLRIKDLTVADSATYYCKALNHCSGEHFDGAGTVLTVNQASGAHHHHHHGAEFEQKLISEED

Supplementary Figure 1

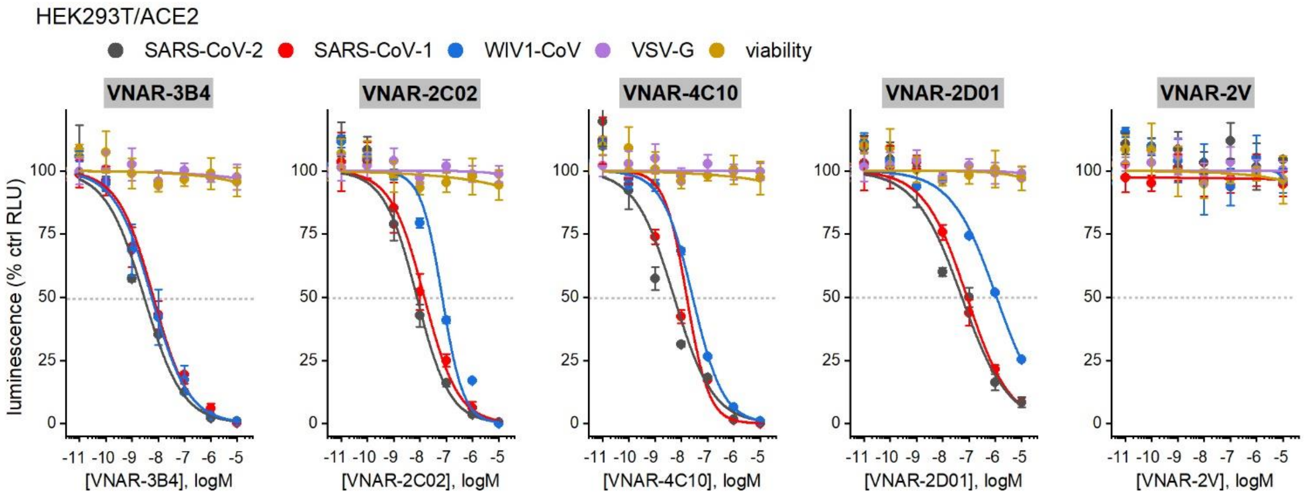

Supplementary Fig. 1 – Antiviral activity of VNAR antibodies in HEK293T-hACE2 cells. Secondary validation of selected neutralizing VNAR domains. Concentration-dependent neutralization of viral particles pseudotyped with glycoproteins natively encoded by either SARS-CoV-2 (black), SARS-CoV-1 (red), WIV1-CoV (blue), or VSV (purple) in HEK293T-hACE2 cells. Cell viability was also assessed in the presence of increasing concentrations of VNARs (yellow). Data represents mean ± s.e.m. RLU values from 3 independent experiments.

Supplementary Figure 2

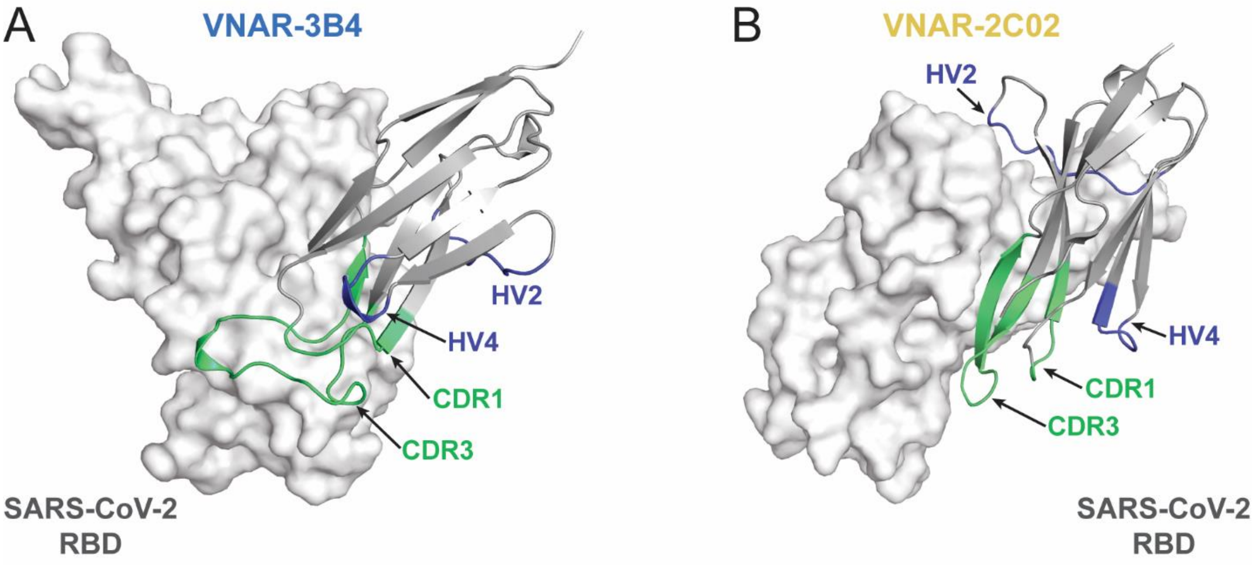

Supplementary Fig. 2 – Structural identification of relevant VNAR regions. A) Figure shows a surface representation of the SARS-CoV-2 RBD with a cartoon representation of VNAR-3B4. The hyper variable regions (HV) are shown in blue and the complimentary determining regions (CDR) are shown in green. **B)** Shown is similar schematic of VNAR-2C02 bound to the RBD.

Supplementary Figure 3

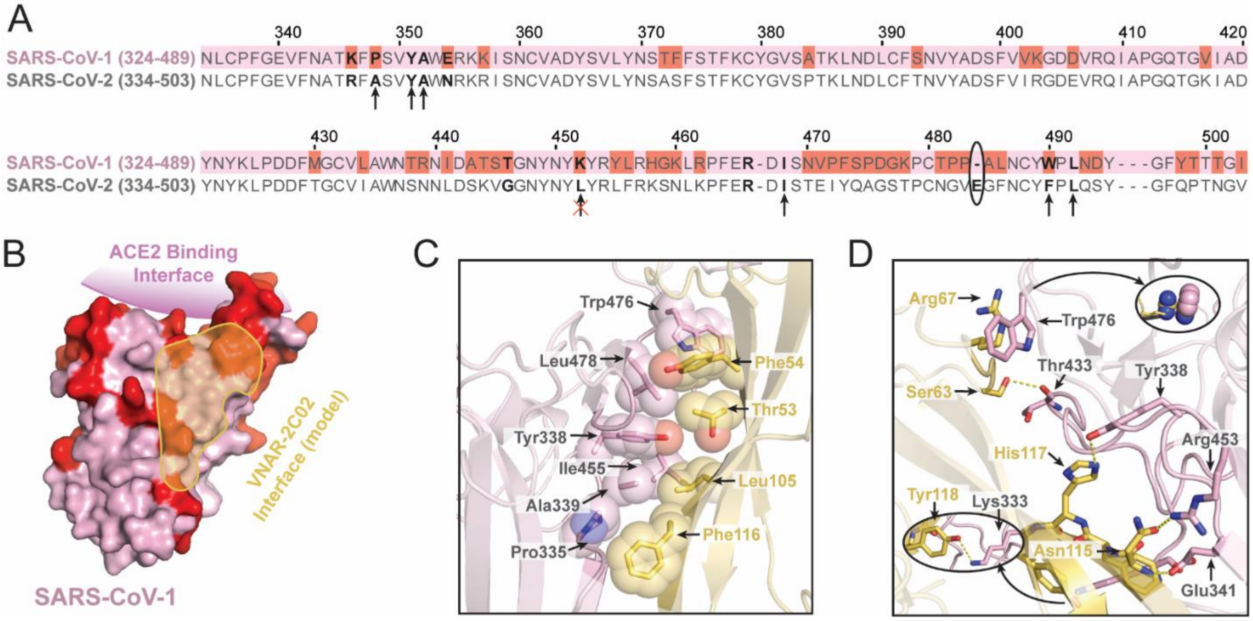

Supplementary Fig. 3 – VNAR-2C02 binds coronaviruses that are closely-related to SARS-CoV-2. A) Shown is a sequence alignment between SARS-CoV-1 and SARS-CoV-2. Residues that are different from SARS-CoV-2 are highlighted in red. Bolded letters indicate residues which are critical for the interaction between the RBD and 3B4 with arrows indicating the residues which form the hydrophobic core of the interaction. The circle annotation indicates a deletion of a residue and the red X indicates a loss of hydrophobicity. Sequence is numbered above according to the SARS-CoV-2 sequence. **B)** Figure shows a surface representation of SARS-CoV-1 (pink) with variant residues colored red and the ACE2 binding interface is highlighted in purple. The homology-modeled interaction interface for 2C02 is colored yellow. **C)** Shown is a zoomed in view of the modeled interaction interface between 2C02 (yellow) and SARS-CoV-1 (pink). Hydrophobic interactions are highlighted as in Figure 4C with residues annotated according to the SARS-CoV-1 sequence. **D)** Shown is a 180-degree view of the modeled interaction interface between 2C02 (yellow) and SARS-CoV-1 (pink). Interactions between residues are drawn as in Figure 4D with residues annotated according to the SARS-CoV-1 sequence. Round insets show alternate views of interactions that are partially obscured from view in the main panel.

Supplementary Figure 4

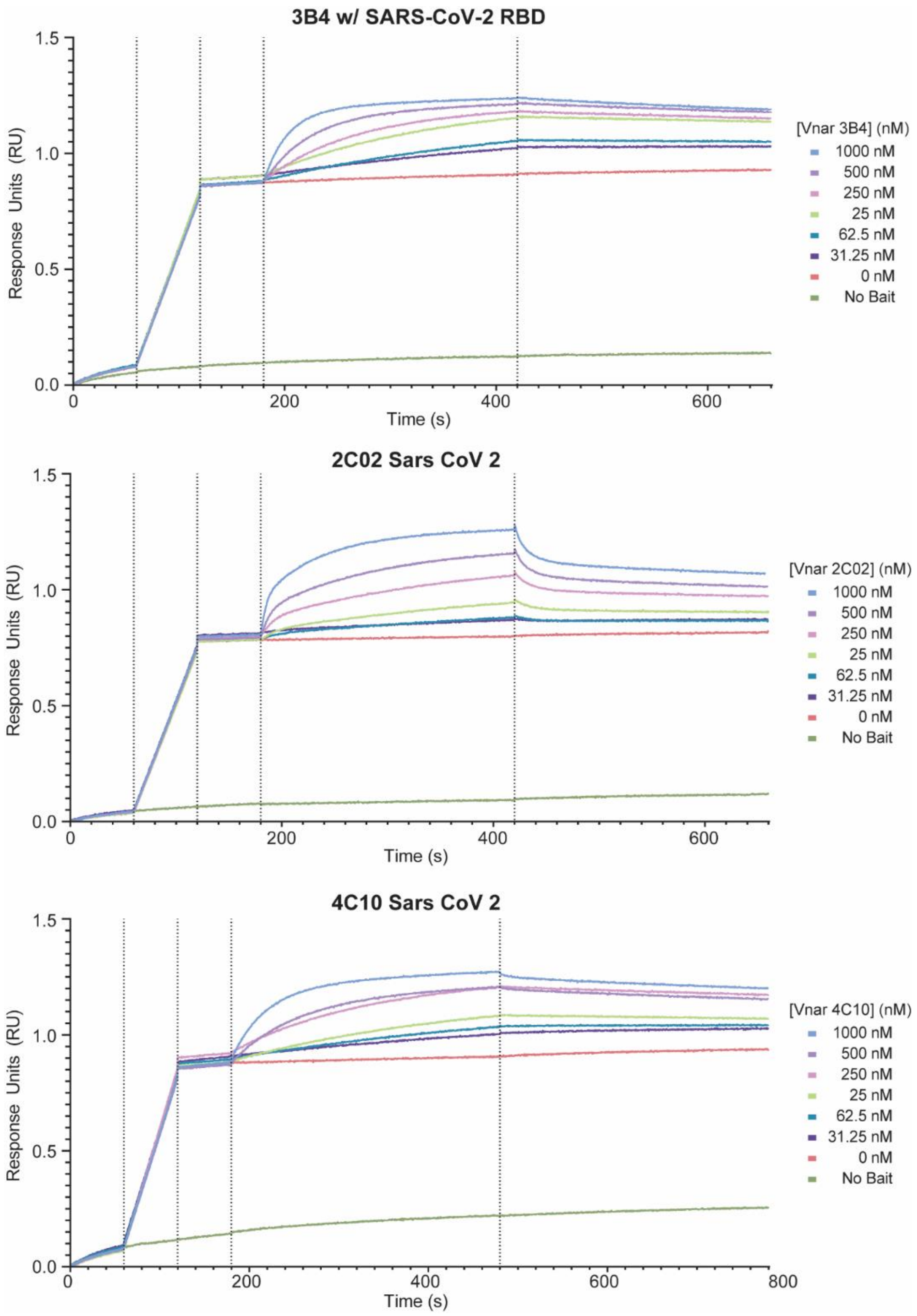

Supplementary Fig. 4 – Biolayer-Layer Interferometry (BLI) binding curves for the VNARs. Dissociation constants for the three VNARs (4C10, 2C02, and 3B4) were determined by BLI using biotinylated wild-type SARS-CoV-2 RBD immobilized on a streptavidin coated biosensor. Binding curves document the association of and dissociation of the VNARs to the immobilized RBD at concentrations ranging from 31.25nM to 1000nM.

Supplementary Figure 5

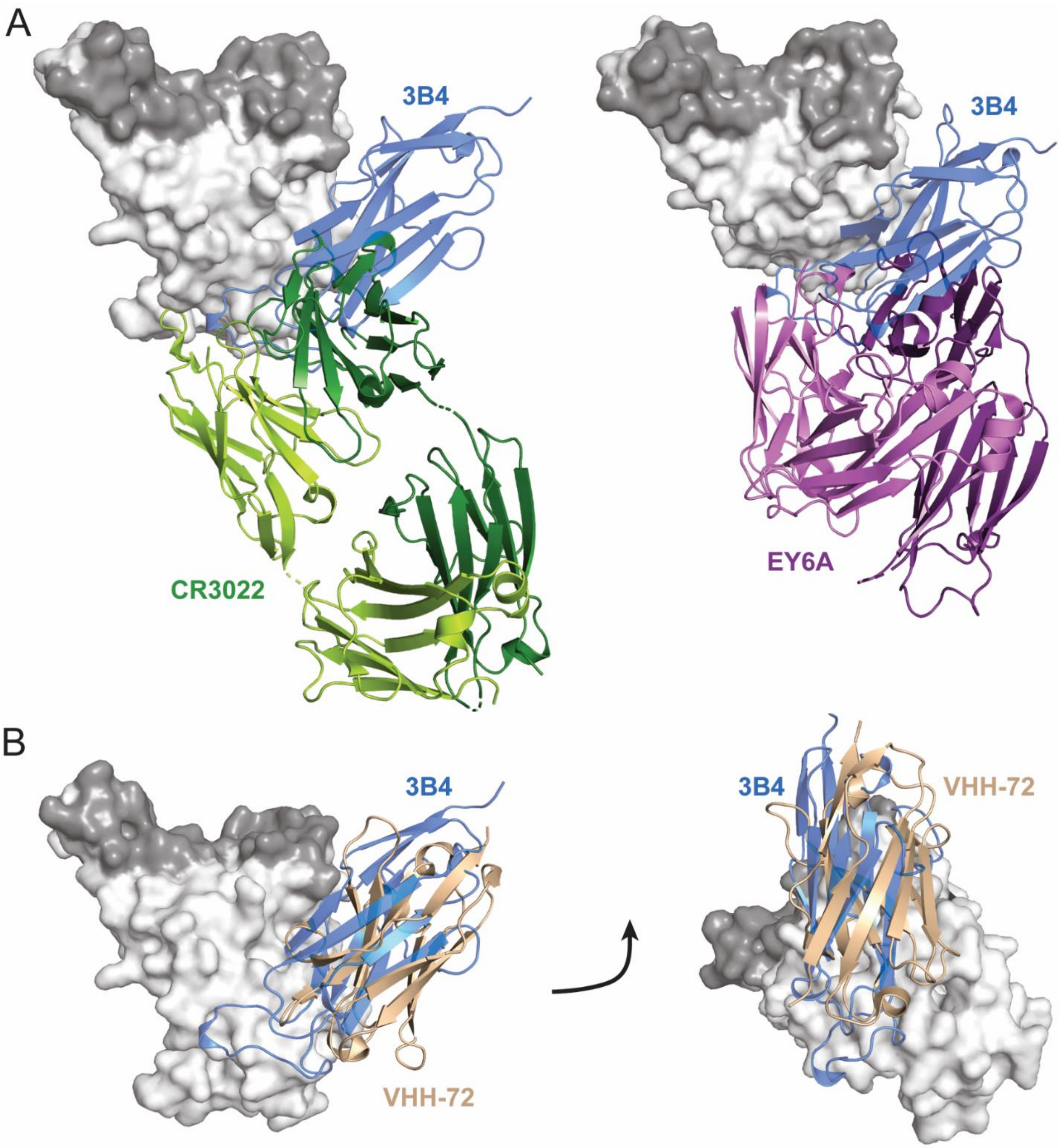

Supplementary Fig. 5 – VNAR 3B4 binds similar to other neutralizing antibodies. A) The SARS-CoV-2 RBD is shown as a gray surface representation with the ACE2 binding site colored dark gray. Neutralizing antibodies are shown bound alongside VNAR 2C02 (yellow). **B)** Shown is a comparison of the overlapping VHH-72 and VNAR 3B4 epitopes from multiple angles with the RBD colored as in Panel A.

Supplementary Figure 6

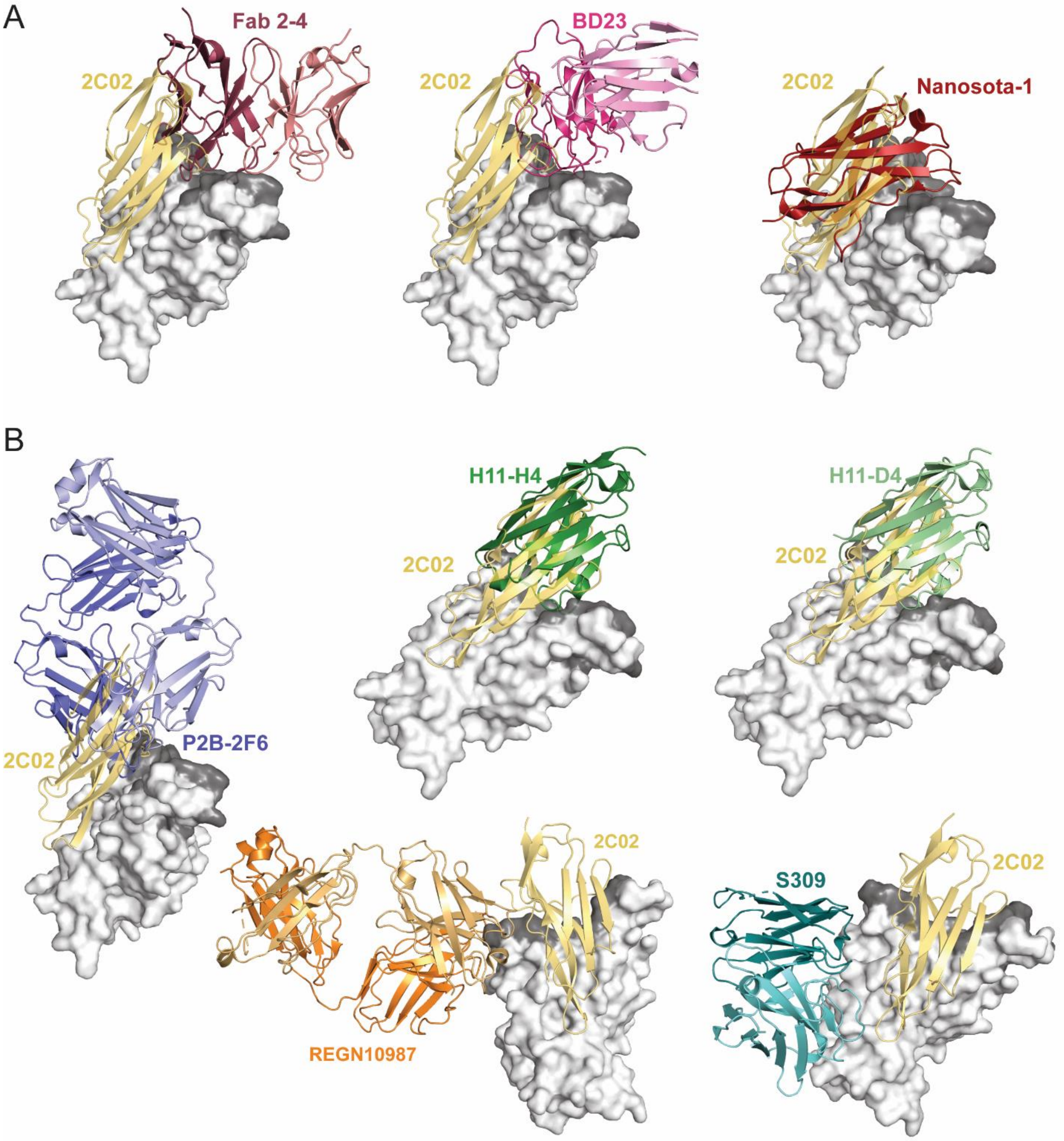

Supplementary Fig. 6 – VNAR-2C02 binds a unique epitope closely resembling other neutralizing antibodies. **A)** The SARS-CoV-2 RBD is shown as a gray surface representation with the ACE2 binding site colored dark gray. Neutralizing antibodies are shown bound alongside VNAR 2C02 (yellow). **B)** Similar structures are shown for other neutralizing antibodies.
