# Supplementary material for: Mechanisms of SARS-CoV-2 neutralization by shark variable new antigen receptors elucidated through X-ray crystallography
Source: Nat Commun. 2021 Dec 16;12:7325. doi: 10.1038/s41467-021-27611-y (PMC8677774; doi:10.1038/s41467-021-27611-y)
Supplement: Supplementary file 2 — Reporting Summary [file 41467_2021_27611_MOESM2_ESM.pdf]

nature portfolio

Corresponding author(s): Aaron M. LeBeau, Hideki Aihara, Caroline J. Barelle

Last updated by author(s): 11/22/21

Reporting Summary

Nature Portfolio wishes to improve the reproducibility of the work that we publish. This form provides structure for consistency and transparency in reporting. For further information on Nature Portfolio policies, see our Editorial Policies and the Editorial Policy Checklist.

Statistics

For all statistical analyses, confirm that the following items are present in the figure legend, table legend, main text, or Methods section.

n/a	Confirmed	
☐	☒	The exact sample size (*n*) for each experimental group/condition, given as a discrete number and unit of measurement
☐	☒	A statement on whether measurements were taken from distinct samples or whether the same sample was measured repeatedly
☒	☐	The statistical test(s) used AND whether they are one- or two-sided<br>*Only common tests should be described solely by name; describe more complex techniques in the Methods section.*
☒	☐	A description of all covariates tested
☒	☐	A description of any assumptions or corrections, such as tests of normality and adjustment for multiple comparisons
☐	☒	A full description of the statistical parameters including central tendency (e.g. means) or other basic estimates (e.g. regression coefficient) AND variation (e.g. standard deviation) or associated estimates of uncertainty (e.g. confidence intervals)
☒	☐	For null hypothesis testing, the test statistic (e.g. *F*, *t*, *r*) with confidence intervals, effect sizes, degrees of freedom and *P* value noted<br>*Give P values as exact values whenever suitable.*
☒	☐	For Bayesian analysis, information on the choice of priors and Markov chain Monte Carlo settings
☐	☒	For hierarchical and complex designs, identification of the appropriate level for tests and full reporting of outcomes
☒	☐	Estimates of effect sizes (e.g. Cohen's *d*, Pearson's *r*), indicating how they were calculated

Our web collection on statistics for biologists contains articles on many of the points above.

Software and code

Policy information about availability of computer code

Data collection	Pseudovirus infectivity data was collected using a Tecan M1000 Pro plate reader with Magellan Standard v7.2, live virus was data was collected using Synergy H1 plate reader from BioTek with Gen5 v2.09.1 software. Bio-layer interferometry data was collected using an Octet Red96E with Octet DataAnalysis v12.0.2.3 software.
Data analysis	Raw virus infectivity data was processed in Microsoft Excel v2110 as described in the Methods section, concentration-response curves and IC50 values were generated using OriginLab v2021b. BLI data was processed in GraphPad Prism v9.0. X-ray crystallography data was processed using XDS version February 5, 2021, PHASER v2.8, COOT v7766, and PHENIX v1.19.2-4158. Homology models were analyzed using PyMol version 2.5

For manuscripts utilizing custom algorithms or software that are central to the research but not yet described in published literature, software must be made available to editors and reviewers. We strongly encourage code deposition in a community repository (e.g. GitHub). See the Nature Portfolio guidelines for submitting code & software for further information.

Data

Policy information about availability of data

All manuscripts must include a data availability statement. This statement should provide the following information, where applicable:

- Accession codes, unique identifiers, or web links for publicly available datasets
- A description of any restrictions on data availability
- For clinical datasets or third party data, please ensure that the statement adheres to our policy

All protein structures are publicly available on RCSB Protein Data Bank. Structures of SARS-CoV-2 RBD in complex with VNAR 3B4 or VNAR 2C02 are original to this work and can be found with accession codes 7SPO and 7SPP, respectively. All other structures were retrieved from PDB, using the following accession codes - 2AJF,

4L72, 6YM0, 4HGK, 7DF4, and 7BF4. All plasmids were sourced as indicated in the Methods. No data availability restrictions will be made, all source data is provided with this paper.

Field-specific reporting

Please select the one below that is the best fit for your research. If you are not sure, read the appropriate sections before making your selection.

- [x] Life sciences
- [ ] Behavioural & social sciences
- [ ] Ecological, evolutionary & environmental sciences

For a reference copy of the document with all sections, see nature.com/documents/nr-reporting-summary-flat.pdf

Life sciences study design

All studies must disclose on these points even when the disclosure is negative.

Sample size	No calculations were made to determine sample sizes. A sample size of 2-3 independent technical replicates (wells) per condition was used for each of n=3 independent biological replicates per condition. This was determined to be sufficient due to the significant and reproducible differences observed between VNAR samples and controls. This sample size also sufficiently produced cross-confirmatory effects in discrete assays, including ELISA, pseudovirus infectivity in multiple cell lines, authentic virus infectivity, and biolayer interferometry. This sample size is noted to be consistent with similar literature published in Nature branded journals within the last year.
Data exclusions	No data exclusions were made.
Replication	Biological experiments were replicated at least 3 times. All attempts at replication were successful, no data was excluded from analyses. All pseudovirus experiments were conducted with 3 technical replicates per condition and n=3 biological replicates per condition. Authentic SARS-CoV-2 infectivity experiments were conducted with 2 technical replicates per condition and n=3 biological replicates per condition.
Randomization	Not applicable to present the study.
Blinding	Blinding was not performed during VNAR screening in ELISA or pseudovirus experiments, as all data represent quantitative measurements that do not require subjective interpretation. Investigators conducting authentic SARS-CoV-2 infectivity assays were blinded to the relative potency of lead antiviral VNARs. No blinding was done with X-ray crystallography experiments due to the nature of structural studies

Reporting for specific materials, systems and methods

We require information from authors about some types of materials, experimental systems and methods used in many studies. Here, indicate whether each material, system or method listed is relevant to your study. If you are not sure if a list item applies to your research, read the appropriate section before selecting a response.

Materials & experimental systems

n/a	Involved in the study
[ ]	[x] Antibodies
[ ]	[x] Eukaryotic cell lines
[x]	[ ] Palaeontology and archaeology
[x]	[ ] Animals and other organisms
[x]	[ ] Human research participants
[x]	[ ] Clinical data
[x]	[ ] Dual use research of concern

Methods

n/a	Involved in the study
[x]	[ ] ChIP-seq
[x]	[ ] Flow cytometry
[x]	[ ] MRI-based neuroimaging

Antibodies

Antibodies used	2 commercial antibodies were used, information as follows:<br>- anti-M13 bacteriophage antibody (clone MM05) was sourced from Sino Biological (catalog # 11973-MM05T-H). The manufacturer website presents data validating usage of this antibody for the application of ELISA detection of antibodies displayed by M13 bacteriophage, just as the antibody was used in the present work.<br>- anti-SARS-CoV-2 Spike antibody (CR3022-RB) was sourced from GeneTex (catalog # GTX01556). This antibody was used as a positive control for detection of SARS-CoV-2 RBD in ELISAs. The manufacturer website confirms usage of this antibody for detection of SARS-CoV-2 RBD in ELISA experiments. Data from the original work describing this antibody (10.1080/22221751.2020.1729069) also demonstrates usage of this antibody in ELISAs.<br><br>Anti-HSA E06 antibody was produced in house and was used for the detection of human serum albumin in ELISA experimens. This antibody was previously characterized and validated in prior publications, and was used for the same application of detecting human serum albumin in ELISA experiments (https://doi.org/10.3389/fimmu.2017.01361 , https://doi.org/10.4161/mabs.22242).

Validation	All commercially available antibodies are widely used and were validated by the manufacturers. Novel antibodies reported in this manuscript are validated in this work.

Eukaryotic cell lines

Policy information about cell lines

Cell line source(s)	HEK293T, Calu-3, and Vero E6 cells were all sourced from ATCC. HEK293T cells stably expressing ACE2 were sourced from BEI resources (NR-52511)
Authentication	All cell lines are authenticated by STR testing, conducted by ATCC.
Mycoplasma contamination	All cell lines are mycoplasma negative
Commonly misidentified lines (See ICLAC register)	HEK293T cells were used, which was used due to high transfection efficiency for the production of virus stocks.
